# Mount Vernon Hospital

**Published:** 1905-07-08

**Authors:** 


### MOUNT VERNON HOSPITAL.

### CHARMING GARDEN PARTY.

A very pleasant garden party was given on Tuesday afternoon by the Mount Vernon Hospital for Consumption at Northwood. Her Royal Highness Princess Christian, who, accompanied by Lord and Lady Ebury, with whom she had been staying, arrived at the hospital in a motor car, and was received by the Marquis of Zetland, President—who thanked her Royal Highness for honouring the entertainment by her presence—by Mr. H. Stedall, Chairman, and the members of the Committee of Management and the hon. medical staff of the hospital. The Princess then personally inspected the wards, etc., and beautiful grounds of the hospital, and spoke to the majority of patients with kindly words of sympathy and interest. Those inmates who were well enough were all seated in comfortable deck chairs on the large balcony extending from the ground-floor wards. Most of them looked brown and bright and strong. The hospital contains 114 beds, but at present, as the condition of the funds requires economy, only 60 are occupied—30 by men, and 30 by women. The staff consists of the matron and 12 other members; probationers are taken for two years' training. The chief event of the fête on Tuesday consisted in the dedication of the new hospital chapel by the Bishop of Islington. During the course of his address the Bishop said how thankful they should all be that the same great care and skill had been expended upon the erection of their chapel as upon that of their grand hospital—a building which was replete with all modern scientific improvements for the relief of suffering. As he himself and all those connected with hospital work well knew, a chapel was a most necessary adjunct to a hospital. The entire fête was most successful and delightful throughout, and should certainly help to win attention and interest for the hospital.

